# The prognostic and predictive value of peripheral immune-related proteins in patients with lung cancer treated with radiotherapy

**DOI:** 10.3389/fonc.2025.1625212

**Published:** 2025-07-17

**Authors:** Shaowen Lyu, Rianne D. W. Vaes, Iris E. W. G. Laven, Francesco Cortiula, Lizza E. L. Hendriks, Marc A. Vooijs, Dirk K. M. De Ruysscher

**Affiliations:** ^1^ Department of Radiation Oncology (Maastro), GROW Research Institute for Oncology and Reproduction, Maastricht University Medical Center+, Maastricht, Netherlands; ^2^ Department of Medical Oncology, University Hospital of Udine, Udine, Italy; ^3^ Department of Pulmonary Diseases, GROW Research Institute for Oncology and Reproduction, Maastricht University Medical Centre+, Maastricht, Netherlands

**Keywords:** lung cancer, radiotherapy, biomarkers, prognostic, predictive, peripheral blood, immune-related proteins

## Abstract

Lung cancer is the leading cause of cancer-related death world-wide. Although the standard of care for patients with advanced stage lung cancer has significantly improved with the advent of immunotherapy and targeted agents, the overall prognosis remains poor. It highlights the need for improved patient selection utilizing prognostic and predictive biomarkers. Given the limited feasibility of serial lung tumor tissue biopsies, liquid biopsies have gained specific interest in achieving this aim. Radiotherapy, commonly used alongside systemic treatments, can induce the release of immuno-stimulatory and immuno-suppressive molecules, triggering the immune- and inflammatory responses and releasing associated molecules. This review specifically focusses on immune-related molecules that are measurable in the blood and which have potential prognostic and/or predictive value in patients with lung cancer treated with radiotherapy alone or in combination with systemic agents. Such immune-related molecules include cytokines and chemokines, damage-associated molecular patterns, soluble receptors and ligands, and proteins expressed on the immune cell surface of circulating immune cells. Classical cytokines IL-6, IL-8, and TGF-β1 were the most studied molecules in patients with lung cancer treated with radiotherapy and were associated with poor survival and increased risk of radiation-induced toxicity. To date, there are still some barriers before these promising findings can be implemented in regular clinical practice. Practical points to achieve this goal are also addressed in this review.

## 1 Introduction

Lung cancer remains one of the leading causes of cancer-related deaths worldwide, with a 5-year overall survival (OS) rate of only 25% (1). Depending on the type of lung cancer, the stage of the disease, and the overall health of the patient, treatment options can include surgery, radiotherapy (RT), chemotherapy, immunotherapy, targeted therapy or a combination of these (2–6). RT is a treatment modality that can be used in all stages of lung cancer. In recent years, both technological advancements (*e.g.*, intensity-modulated RT) and the integration of immunotherapy have broadened the indication for RT and have improved patients’ outcomes by reducing RT-related toxicity and increasing OS, respectively (7, 8). However, in patients with lung cancer, radio-resistance and radiation-induced toxicity are both significant contributors to RT failure, resulting in cancer progression and deterioration of quality of life (QoL) (9).

Whereas research has mainly focused on the level and mechanisms of RT-induced DNA damage, and the repair capacity of irradiated cancer cells, the field has largely ignored the fact that the radiosensitivity of cancer cells in patients is greatly affected by the immunocompetence of the host (10). RT not only induces DNA double-strand breaks followed by some forms of cell death (*i.e*. apoptosis, necrosis, mitotic catastrophe, or replicative senescence) but it can also induce clinically relevant tumor-targeting immune responses, which critically rely on the host’s immune status and the antigenicity of cancer cells and their capacity to generate adjuvant signals (11, 12). Upon RT-induced DNA damage, DNA accumulation in the cytoplasm of irradiated cells can be sensed by cytoplasmic nucleic acid sensors, resulting in activation of the cyclic GMP-AMP synthase-simulator of interferon genes (cGAS-STING) pathways which in turn lead to a systematically interferon type I (IFN-I) driven immunity program (13, 14). Besides, RT can induce an immunogenic variant of tumor cell death (ICD), which is accompanied by the expression and release of damage-associated molecular patterns (DAMPs). These RT-induced immunogenic responses can result in the uptake of tumor-associated antigens (TAAs) by dendritic cells (DCs) that present them to cytotoxic CD8+ T-cells recruited from circulatory system, subsequently priming and activating the anti-tumor immunity response (15).

In contrast to these RT-induced immunostimulatory effects, RT can also induce immunosuppressive responses, such as the secretion of immune suppressive cytokines (*i.e.* granulocyte-macrophage colony-stimulating factor, GM-CSF; transforming growth factor-β, TGF-β) from tumor cells, which promote the migration of myeloid-derived suppressor cells (MDSCs) and regulatory T cells (Treg) from the circulation towards irradiated areas (16). Also, there is increasing evidence showing that irradiation can induce the expression of the immune checkpoint programmed death ligand 1 (PD-L1) on tumor cells. PD-L1 can induce T-cell anergy (*i.e.*, absence of the normal immune response to a particular antigen or allergen) or apoptosis by binding to PD-1 on activated T-cells, and thereby prevent the killing of cancer cells (17, 18).

Besides the radiosensitivity and immunogenicity of tumor cells itself, the tumor microenvironment (TME) can shift from an immunosuppressive “cold” TME to an immunostimulatory “hot” TME and vice versa (19). An immune-desert TME is generally characterized by high numbers of MDSCs and Tregs and enrichment of immunosuppressive cytokines, such as TGF-β and interleukin-10 (IL-10). An immune-enriched TME is characterized by high PD-L1 expression on the tumor cell surface, high number of effector immune cells (*e.g.*, CD8+ T-cells, natural killer cells), and immunostimulatory cytokines and chemokines (*i.e*. interferons). The switch from a “cold” towards a “hot” tumor highly depends on the local production of cytokines, chemokines and other soluble factors but also on the trafficking or modulation of immune cell subsets recruited from circulatory system into the TME (19). This transition may be impaired by intrinsic tumor cell radio-resistance mechanisms, tumor heterogeneity, resistance-promoting microenvironment and immunocompetence of the host, resulting in a proportion of patients that initially do or do not respond to RT (10).

Another key aspect limiting the success of RT for lung cancer treatment is RT-induced lung injury (RILI), which encompasses any lung toxicity induced by RT and can manifest acutely – in an early phase – as radiation pneumonitis and in a late phase as radiation pulmonary fibrosis resulting from chronic pulmonary tissue damage (8, 20). Acute radiation damage induced on endothelial and epithelial cells mainly includes DNA damage and release of reactive oxygen species (ROS), which cause cell death, release of pro-inflammatory proteins like CXC-chemokine ligand 12 (CXCL12), interleukin-1 (IL-1), recruiting immune cells to irradiated areas, edema of the alveolar walls and increased vascular disruption (21). Protracted fractions of irradiation can cause endothelial cell dysfunction, leading to increased membrane permeability, detachment and apoptosis, activation and release of inflammatory cytokines like TGF-β, eventually leading to chronic radiation-induced pulmonary fibrosis (22, 23). Almost one out of four patients (24%) with stage II-III non-small-cell lung cancer (NSCLC) treated with chemoradiotherapy experience RILI with grade 2 or more (24, 25), underlining the need for predictive and prognostic biomarkers.

Tissue biopsies are the golden standard procedure for diagnosis and molecular testing, however, a tissue biopsy is an invasive method that is not always feasible and repeatable. Also, the diagnostic information obtained from these tumor biopsies is only used for treatment selection, without providing any additional information regarding radio-resistance or the development of RILI (26). Moreover, tissue biopsies can only provide information limited to a single timepoint and a single tumor location. Tissue biopsies are also unable to capture the dynamic changes of the tissue during treatment. In recent years, liquid biopsies have gained interest due to their non-invasive, cost-effective nature and the ability to be repeatedly collected. Peripheral blood is the main source of circulating immune-related molecules and therefore may be a valuable surrogate for the actual systemic immune status of patients’ before, during, and after treatment. To date, there is limited understanding of whether the immunocompetence of the host and the RT-induced immunological changes can be captured systemically in the blood, and whether these features may serve as prognostic or predictive markers for treatment outcome. As such, there is an urgent need for liquid prognostic and predictive biomarkers that can aid in patient selection and monitoring treatment outcomes for patients with lung cancer treated with RT. This review aims to summarize and discuss the current literature regarding peripheral immune-related proteins that are associated with treatment responses and treatment outcome in patients with lung cancer treated with RT alone or in combination with other systemic anti-cancer medications. Clinically relevant immunological prognostic and predictive biomarkers can help clinicians to timely identify the patients with a poor prognosis or the patients who are at high risk to develop RT-induced toxicity.

## 2 Prognostic and predictive peripheral immune biomarkers

RT can exert immunostimulatory and immunosuppressive effects, both locally, within the irradiated TME, and systemically, outside the radiation field (27). Molecules involved in these RT-activated immunological signaling cascades include cytokines, chemokines, damage-associated molecular patterns (DAMPs), soluble proteins such as soluble receptors and ligands, and soluble forms of immune cell surface proteins. The circulating levels of these proteins and their fluctuations before, during and after treatment may represent the actual immunological status of patients. This information may aid clinicians to predict the patient’s prognosis and tailor treatments.

To date, numerous clinical trials have investigated the prognostic and predictive significance of these circulating immune-related proteins in the blood of patients with lung cancer treated with RT, as summarized in **Tables 1**, **2** and discussed in detail below. Prognostic biomarkers are defined as biomarkers that provide information about the oncological outcome, regardless of treatment, whereas predictive biomarkers indicate the probability of a therapeutic benefit from a specific therapy.

**Table 1:** The prognostic and predictive value of circulating immune-related biomarkers in relation to treatment outcome in patients with lung cancer treated with radiotherapy.

Biomarkers	Number of patients	Treatment	Prognostic or predictive	Relationship		Reference
Cytokines						
IL-6	41	(Chemo)RT	Predictive	↑ IL-6	↓ OS	Gkika et al. (28)
	23	RT + ICB	Predictive	↓ IL-6	↑ PFS/OS	Ye et al. (29)
TGF-β	82	(C)RT	Predictive	↓ TGF-β	↑ Effective treatment response	Luo et al. (30)
	65	(C)RT	Predictive	↓ TGF-β	↑ PFS/OS	Zhao et al. (31)
	58	RT	Predictive	↓ TGF-β	↑ Effective treatment response	Fu et al. (32)
IL-8	58	RT	Predictive	↓ TGF-β	↑ Effective treatment response	Fu et al. (32)
	41	(C)RT	Predictive	↑ IL-8	↓ OS	Gkika et al. (28)
	45	(C)RT	Predictive	↑ IL-8	↑ MTV	Eide et al. (33)
	44	(C)RT + ICB	Predictive	↑ IL-8	↓ OS	Kang et al. (34)
	49	RT + ICB + GM-CSF	Prognostic/Predictive	↑ IL-8	↑ PFS/OS	Ni et al. (35)
IL-1	19	CRT	Prognostic	↑ IL-1	↑ OS	Tang et al. (36)
IL-10	26	RT	Prognostic/Predictive	↑ IL-10	↓ PFS	Vaes et al. (37)
	23	RT + ICB	Predictive	↓ IL-10	↑ PFS/OS	Ye et al. (29)
OPN	337	(C)RT	Prognostic	↓ OPN	↑ OS	Suwinski et al. (38)
	55	(C)RT	Predictive	↓ OPN	↑ FFR	Ostheimer et al. (39)
IP-10/CXCL10	26	RT	Predictive	↑ IP-10	↓ PFS	Vaes et al. (37)
GM-CSF	72	(C)RT	Predictive	↑ GM-CSF	↑ PFS/OS	Deng et al. (40)
IFN-β	35	RT + ICB	Predictive	↑ IFN-β	↑ Effective treatment response	Formenti et al. (41)
IL-17A	23	RT + ICB	Predictive	↓ IL-17A	↑ PFS/OS	Ye et al. (29)
IL-21	51	RT + ICB + GM-CSF	Predictive	↑ IL-21	↑ PFS/OS	Ni et al. (35)
Soluble receptors and ligands						
sTNFR1	62	CRT	Predictive	↑ sTNFR1	↑ Symptom burden	Wang et al. (42)
sIL-2R	181	(C)RT	Prognostic	↑ sIL-2R	↓ OS	Carvalho et al. (43)
sPD-L1	126	CRT	Prognostic	↑ sPD-L1	↓ OS	Zhao et al. (44)
	31	(C)RT	Prognostic	↓ sPD-L1	↑ Tumor remission	Sui et al. (45)
sCD244	26	RT	Prognostic	↑ sCD244	↓ PFS	Vaes et al. (37)
sCR2	18	CRT	Prognostic	↑ sCR2	↑ PFS	Vaes et al. (37)
Proteins on immune cell surface						
CD28+CD8+ T-cells	41	RT	Prognostic	↑ CD28+CD8+ T cells	↑ Tumor remission	Liu et al. (46)
TCR repertoire	19	RT	Predictive	↓ Shannon entropy*	↑ Metastasis	Wu et al. (47)
TCR repertoire	15	RT + ICB	Predictive	↓ Shannon entropy*	↑ PFS/OS	Öjlert et al. (48)

*Shannon entropy index is a metric for measuring variable gene expression levels. Treatment response was evaluated according to RECIST 1.1. ↑, higher level of molecule; ↓, lower level of molecule; CRT, chemoradiotherapy; ICB, immune checkpoint blocker; MTV, metabolic tumor volume; NR, not reported; OS, overall survival; PFS, progression-free survival; RT, radiotherapy.

**Table 2:** The prognostic and predictive value of circulating immune-related biomarkers in relation to the development of RILI (GR≥2) in patients with lung cancer treated with radiotherapy.

Biomarkers	Number of patients		Treatment	Prognostic or predictive	Relationship		Reference
	Total cohort (*N*)	RILI (*N*)					
Cytokines							
IL-6	432	NR	NR	Prognostic	↑ IL-6	↑ RILI	Fu et al. (49)
	15	6	CRT	Predictive	↑ IL-6	↑ RILI	Jeong et al. (50)
	24	13	(C)RT	Prognostic/predictive	↑ IL-6	↑ RILI	Chen et al. (51)
TGF-β	142	29	(C)RT	Predictive	↑ TGF-β	↑ RILI	Wang et al. (52)
	80	10	CRT	Predictive	↑ TGF-β	↑ RILI	Liu et al. (53)
	34	8	Surgery + RT/(C)RT	Predictive	↑ TGF-β	↑ RILI	Kim et al. (54)
	165	29	(C)RT	Predictive	↑ TGF-β	↑ RILI	Zhao et al. (55)
IL-8	142	29	(C)RT	Prognostic/predictive	↓ IL-8	↑ RILI	Wang et al. (52)
IL-1	24	13	(C)RT	Prognostic/predictive	↑ IL-1	↑ RILI	Chen et al. (51)
IL-10	96	7	Surgery + RT/(C)RT	Predictive	↓ IL-10	↑ RILI	Arpin et al. (56)
TNF-α	104	21	Surgery + RT/(C)RT	Prognostic	↑ TNF-α	↑ RILI	Li et al. (57)
IP-10/CXCL10	12	5	(C)RT	Predictive	↓ IP-10	↑ RILI	Siva et al. (58)
Soluble receptors and ligands							
sTNFR1	39	8	CRT	Predictive	↓ sTNFR1	↑ RILI	Hinton et al. (59)
Proteins on immune cell surface							
CD57+CD28-CD8+ T-cells	54	11	CRT	Prognostic/predictive	↑ CD57+CD28-CD8+ T-cells	↑ RILI	Kim et al. (60)

↑, higher level of molecule; ↓, lower level of molecule; CRT, chemoradiotherapy; NR, not reported; RILI, radiation-induced lung injury; RT, radiotherapy.

### 2.1 Cytokines

Cytokines are soluble polypeptides that mediate cell-to-cell communication, functioning as chemical messengers in the human body. They are produced and secreted by many different cell types, including immune cells, epithelial cells, and endothelial cells that are present in the TME and healthy surrounding tissue. There are different subclasses of cytokines, including interleukins, chemokines, interferons, and tumor necrosis factors (TNF), which all play an important role in regulating inflammatory responses. These cytokines are often produced in a cascade as one cytokine stimulates its target cells to produce and secrete additional cytokines, and increases in hundreds fold in response to the injury (61).

Irradiation can induce acute responses in the irradiated tissue, resulting in the production of numerous inflammation-related cytokines such as IL-1, IL-6, IL-8, and TNF-α within minutes to hours (62). These pro-inflammatory cytokines are instrumental in generating free radicals and oxidative stress, leading to secondary DNA damage, inflammation, and potentially RILI (63, 64), whereas anti-inflammatory cytokines, such as IL-10 and TGF-β show anti-oxidative properties (65, 66). Also, RT-induced tissue damage results in the expression and/or release of DAMPs in a time-dependent manner. Whereas ATP is secreted rapidly after irradiation, others are secreted hours to days after RT mainly by dead cells (22, 67).

#### 2.1.1 Interleukin-6

IL-6 is a multifunctional cytokine that is produced by many cell types like tumor cells, immune cells, and smooth muscles that play important roles during inflammation and immune responses (68). IL-6 is involved in the acute inflammation process by inducing the production of C-reactive proteins and serum amyloid A (SAA), but also by reducing serum iron levels resulting in anemia. Furthermore, IL-6 is recognized as a key regulator of immunosuppression in patients with advanced cancer (69, 70). Blockade of IL-6 in mice has been shown to significantly inhibit lung cancer progression, tumor cell–intrinsic STAT3 activation, tumor cell proliferation, and angiogenesis (71).

In pre-clinical studies, IL-6 levels have been shown to increase after irradiation and predominantly exert immunosuppressive functions. For example, Ao et al. showed that serum IL-6 levels were already increased 6 hours after irradiation in mice (72). Similarly, Xin et al. showed that IL-6 levels significantly increased within 24 hours after irradiation, mediating macrophage infiltration and promoting tumor metastasis in mice (73).

In the clinical setting, numerous studies have evaluated the role of circulating IL-6 in patients with lung cancer treated with RT. For example, in patients with histologically proven thoracic malignancies, Gkika et al. showed that circulating levels of IL-6 at the end of RT and during follow-up were inversely correlated with OS (28), as well as Ye and associates for both PFS and OS (29). A meta-analysis performed by Fu and colleagues revealed that patients with RILI had significantly higher serum IL-6 levels before RT than those without RILI (49). Similar findings have been reported by numerous others (42, 50, 51, 74). Furthermore, besides high IL-6 levels before RT, increased IL-6 levels during and after RT also appeared to be associated with the development of RILI. For instance, Jeong et al. showed that IL-6 levels peaked at week 3 after RT initiation in patients who developed RILI (50). Chen and associates showed that absolute levels of IL-6 were significantly higher before, during and after RT in patients who developed RILI (51). These findings suggest that IL-6 could be a promising biomarker for developing RILI.

#### 2.1.2 Transforming growth factor-β

TGF-β is a master regulator of cellular proliferation and tissue homeostasis (75). There are three known isoforms of TGF-β, of which TGF-β1 is the most abundant and ubiquitously expressed (76). In cancer, TGF-β plays dual roles as it can exert tumor suppressor effects on normal healthy cells and early carcinogenesis by regulating cell growth and apoptosis. However, during tumor development, these tumor suppressor effects are often lost and then switches to promote cancer progression, invasion, and tumor metastasis (77). In the TME, TGF-β can promote suppression of the anti-tumor immunity by inducing polarization of macrophages towards the M2 anti-inflammatory phenotype, inhibiting the release of IL-2 by naïve T cells to prevent proliferation of cytotoxic T-lymphocytes (CTLs) and NK cells, and inducing cancer-associated fibroblasts to release interleukin-11 (IL-11) that can increase the metastatic capacity of cancer cells (78–81). Suppression of TGF-βs by antibody-mediated TGF-β neutralization in combination with RT has been shown to increase the numbers of CTLs and NK cells within the TME (82).

Numerous pre-clinical studies have investigated the role of TGF-β during irradiation. *In vivo* studies showed that RT-induced ROS activate TGF-β, which encompasses the release of TGF-β from the latency-associated protein (LAP) that is required for the binding of TGF-β to its receptor (83). Also, the clearance of RT-induced dead tumor cells by macrophages can trigger the release of TGF-β by macrophages (79). Furthermore, TGF-β has been shown to exert multiple functions within irradiated lung cancer cells. On one hand, TGF-β can promote the DNA damage response both *in vitro* and *in vivo* reducing the radiosensitivity of tumor cells (84). On the other hand, TGF-β signaling has been shown to be involved in ionizing radiation-induced fibrosis through both canonical and noncanonical TGF-β pathways (79), either by Smad 2/3 pathway or mir-21 (85), Rho/ROCK (86) and NADPH oxidase (87) in lung.

Given that TGF-β regulates a plethora of cellular responses, numerous clinical trials have investigated its clinical significance in patients with lung cancer. For example, Luo et al. investigated the potential predictive value of TGF-β1 in 82 patients with lung cancer treated with RT (30). Within one week after RT, TGF-β1 levels were significantly decreased in patients who achieved an effective response according to RECIST 1.1 compared to patients who did not. Also, TGF-β1 levels were negatively correlated with circulating CD4+, CD8+ and the CD4+/CD8+ ratio during and at the end of RT (30). In addition, patients who had significantly reduced TGF-β1 levels 2 weeks after initiation of RT compared to pre-RT seemed to have a better treatment response than those who had higher TGF-β1 levels (30). Zhao et al. collected data from patients with NSCLC treated with chemoradiotherapy and showed that 4 weeks after the first fraction of RT, decreased TGF-β levels were significantly associated with a prolonged OS and PFS compared to patients with increased TGF-β1 levels (31). Similarly, Fu et al. showed that in patients with unresectable NSCLC treated with three-dimensional conformal radiation therapy (3D-CRT), TGF-β1 levels were significantly decreased in patients who achieved a radiological response after 3D-CRT (32). Overall, decreased TGF-β1 levels during and after RT seem to be associated with better outcomes.

Besides the association of TGF-β1 with treatment response and outcome, TGF-β1 has also been associated with the development of RILI. Wang et al. showed that a higher mean lung dose and a higher TGF-β1 2w/pre ratio (i.e., TGF-β levels at 2 weeks after RT initiation divided by the TGF-β1 levels before the first fraction of RT) in combination with lower pre-treatment IL-8 levels were associated with a higher risk of developing RILI in patients with NSCLC (52). Similarly, Liu et al. demonstrated that in patients with stage III NSCLC who underwent 3D-CRT, circulating TGF-β1 levels increased during the first 2 weeks after the first fraction of RT and were significantly increased at week 6 in the patients who developed RILI (grade ≥1) (53). Similarly, Kim et al. observed significant associations between the changes of TGF-β1 during the time course of RT and the risk of developing RILI in patients with lung cancer (54). Zhao et al. reported similar results in patients with stage I-III NSCLC (55). They showed that patients with increased TGF-β1 levels 4 weeks after the first fraction of RT are more likely to develop RILI than those who do not. These results were validated by them in a larger cohort (88). In summary, alterations in TGF-β levels during and after RT are predominantly associated with a high risk of developing RILI.

#### 2.1.3 Interleukin-8

Interleukin-8 (IL-8/CXCL8), is a pro-angiogenic and pro-inflammatory chemokine (89). The biological effects of IL-8 are mediated through the binding of IL-8 to two cell-surface G protein–coupled receptors, CXCR1 and CXCR2 (90). Different stimuli can induce the expression and release of IL-8 by various cell types, including inflammatory signals (i.e. TNF-α, IL-1β), chemical and environmental stresses (i.e. RT and hypoxia), and steroid hormones (89, 91). In tissue specimens of patients with lung cancer, high levels of IL-8 have been shown to correlate with tumor stage and prognosis (92, 93). Furthermore, IL-8 has been shown to stimulate tumor cell proliferation and promote angiogenesis by recruiting endothelial cells to the TME (94).

So far, only a limited number of pre-clinical studies have investigated the role of RT on the expression and release of IL-8 and its function in lung cancer models. *In vitro*, RT has been shown to induce IL-8 expression via the p38/MAPK and NF-κB signaling pathways in lung cancer cells (95). *In vivo* studies also showed that stereotactic ablative radiotherapy (SABR) could induce IL-8 secretion by lung cancer cells (96). Furthermore, Kühlmann et al. showed that increased IL-8 levels in the supernatant of irradiated lung epithelial cells could stimulate collagen synthesis and matrix production in lung fibroblasts (97). Accordingly, irradiation may stimulate the secretion of IL-8, which in turn can promote tumor development and lung fibrosis in lung cancer.

Numerous clinical studies have investigated the prognostic and predictive value of IL-8 in patients with lung cancer treated with RT (28, 33–35). In a recent study, Gkika et al. showed that IL-8 levels during and at the end of RT were negatively correlated with OS (28). Eide et al. revealed that IL-8 serum levels before, during and after treatment were all positively correlated with the metabolic tumor volume (i.e. FDG uptake) in patients with advanced NSCLC undergoing palliative RT (33). In 2023, Kang et al. investigated the predictive value of IL-8 in patients with advanced NSCLC who received hypo-fractionated RT combined with PD-1 blockade immunotherapy (34). In this study, high pre-treatment levels of circulating IL-8 were significantly associated with a poor prognosis and 3 months after treatment, a remarkable decrease of IL-8 was only observed in patients in the partial remission group compared to the non-responder group (34). Notwithstanding, Ni and colleagues showed that higher pre-SBRT and post-SBRT levels of circulating IL-8 were prognostic and predictive, respectively, for improved PFS and OS, although not significant (35).

Furthermore, IL-8 has been shown to be a good predictor for developing post-RT toxicities. Wang et al. collected blood samples from 142 patients with stage I-III NSCLC treated with RT and found that low circulating IL-8 levels before and 2–4 weeks during RT were significantly associated with a higher risk of developing RILI (52).

#### 2.1.4 Interleukin-1

The interleukin-1 (IL-1) family, comprising 11 cytokines, plays a central role in innate and acquired immunity (98). Previous studies have mainly focused on interleukin-1α (IL-1α) and interleukin-1β (IL-1β) during irradiation. IL-1α, predominantly produced by mesenchymal cells, is a key cytokine involved in acute inflammatory responses. It can induce the production of other cytokines such as IL-2, IL-6, and TNF-α, and enhance their relative biologic effectivity (99, 100). The main producers of IL-1β are innate immune cells, such as monocytes and macrophages (101). In cancer, IL-1 has been shown to promote carcinogenesis, induce tumor growth, metastasis and exert immunosuppressive functions (102).

Numerous pre-clinical studies have investigated the effects of irradiation on IL-1 expression in lung cancer. In healthy mice lung tissues, RT induces a biphasic expression pattern of IL-1α. An initial rise was observed at 6 hours after irradiation followed by a drop to basal levels at 2 weeks, whereas these levels increased again at 8 weeks due to RT-induced inflammation (103). Interestingly, Johnston et al. showed that IL-1α and IL-1β remained elevated in healthy mouse lungs up to 6 months after irradiation and contributed to the radiation-induced pulmonary fibrosis (104). Kang et al. demonstrated that IL-1β enhances migration and invasion in the A549 NSCLC cells via the NF-κB–RIP1- IL-1β pathway (105).

There are only a limited number of clinical studies that have explored the dynamic changes and potential prognostic or predictive value of IL-1 in patients with lung cancer treated with RT. Trovò et al. reported decreased IL-1 levels in 13 patients with locally advanced NSCLC within 4 weeks following radical moderated hypo-fractionated RT (60 Gy/25 fractions) (106). However, it was not reported whether the decreased IL-1 levels were associated with treatment outcome as the purpose of the study was only to assess the kinetics of plasmatic cytokines during RT. In the NCT01725165 phase II trial, high baseline circulating IL-1α levels were significantly associated with improved outcomes in 19 patients with oligometastatic NSCLC (36). Furthermore, IL-1α has also been identified as a potential predictive biomarker. Chen et al. revealed that in patients with lung cancer (n=24), IL-1α blood levels were significantly higher before, during and after RT in patients who developed RILI (51). In addition, the authors showed that both IL-1α and IL-6 circulating levels gradually increased and were positively correlated with time, especially after RT, which may suggest that both IL-1α and IL-6 are involved in the response to radiation injury (51, 107).

#### 2.1.5 Interleukin-10

IL-10 is a key anti-inflammatory cytokine that modulates inflammation and maintains cell homeostasis. It is mainly produced by monocytes, macrophages, and cytotoxic T-cells and can inhibit the synthesis of pro-inflammatory cytokines like IL-2 and TNF-α, while it also exerts immunostimulatory effects on B-cells, cytotoxic T cells and thymocytes (108–110). An *in vitro* study showed that low-dose irradiation of 4 Gy can induce IL-10 secretion by lung tumor cells 6–48 hours post-irradiation (111). *In vivo*, IL-10 significantly increased from 24 to 96 hours after irradiation. It showed that hypo-fractionated RT could induce the production of IL-10 by CD8+ T-cells, enhancing their proliferation, differentiation, activity, and function (18).

Only a few studies have investigated the clinical significance of IL-10 in patients with lung cancer undergoing RT. We demonstrated that high circulating levels of IL-10 at baseline, during, and end of stereotactic body radiotherapy (SBRT) were significantly associated with worse PFS in patients with stage I NSCLC (n=26) (37). Ye and his colleagues corroborated these results for increased plasma levels of IL-10 following treatment in Nivolumab responders (29). For RILI, Arpin et al. performed a multivariate analysis of serum cytokine levels on patients with NSCLC during the first two weeks of RT (56). Their results indicated that elevated IL-6 and decreased IL-10 levels were associated with a high likelihood of developing RILI .

#### 2.1.6 Other cytokines

In addition to the cytokines mentioned above, several other circulating cytokines have been reported to have prognostic or predictive potential in patients with lung cancer treated with RT. However, the association of these cytokines with patient outcomes has only been reported in a very limited number of clinical studies.

One of these cytokines is osteopontin (OPN), a secreted phosphorylated glycoprotein that is involved in inflammation, tumor progression, and metastasis (112). OPN is activated under hypoxia and OPN concentrations are associated with both tumor hypoxia and outcomes after RT in patients with cancer (113, 114). In 337 patients with NSCLC, Suwinski et al. showed that low OPN concentrations before the start of (chemo)radiation were significantly associated with a favorable OS (38). Ostheimer et al. reported a higher risk of relapse in patients with inoperable NSCLC whose OPN were stable or increased 4 weeks after RT (n=55), indicating that OPN may be associated with a more aggressive cancer phenotype (39, 115). In 2016, Carvalho et al. improved a clinical prognostic model by incorporating OPN (43). The inclusion of OPN significantly improved the discrimination of the model to better predict the prognosis of patients with stage I-IIIB NSCLC treated with RT.

Other immune-related cytokines reported in patients with lung cancer treated with RT include TNF-α (57), IFN-β (41), IFN-γ (37, 116), interferon gamma-induced protein 10 (IP-10/CXCL10) (37), monocyte chemoattractant protein-1 (MCP-1) (58), vascular endothelial growth factor (VEGF) (38), erythropoietin (EPO) (38), GM-CSF (116), IL-17A (29), and IL-21 (35). TNF-α is a pro-inflammatory cytokine which can induce the synthesis and release of other cytokines, including IL-6 and IL-1 (117). TNF-α levels have been shown to increase after RT in patients with lung cancer (n=104), but its relationship with survival was not reported (57). Furthermore, the baseline TNF-α levels were higher in patients who developed RILI (57). Siva et al. showed that patients with stage I-III NSCLC who developed RILI have decreased circulating levels of IP-10/CXCL10, MCP-1 and eotaxin after the first fraction of RT compared to patients without RILI (n=12) (58). Also, Vaes et al. implied that after the first fraction of SBRT, increased IP-10/CXCL10 levels were significantly associated with a shorter PFS for patients with stage I NSCLC (n=26) (37). Deng et al. demonstrated that upregulated GM-CSF during RT correlated with longer OS and PFS in patients with unresectable lung cancer, and it was an independent predictive factor (40), which is in line with its antitumor immune function (118). Lastly, Formenti et al. analyzed the blood samples of patients with metastatic lung cancer that were treated with palliative RT and the anti-CTLA-4 antibody, ipilimumab (n=35) (41). They showed that IFN-β was significantly increased 22 days after completion of RT in patients with partial/complete response and stable disease but not in patients with progressive disease or death.

### 2.2 Damage-associated molecular patterns

Other interesting immune-related molecules that can act as predictive and prognostic biomarkers include DAMPs. Heat shock proteins (HSPs), particularly HSP70 and HSP90 (37, 119), are intracellular chaperones that can act as damage-associated molecular patterns (DAMPs) when exposed on the cell surface or released extracellularly during stress or cell death. In patients with NSCLC treated with immunotherapy and chemotherapy, increased plasma levels of HSP90 at diagnosis has shown to be prognostic for survival (119). In addition, high mobility group box 1 (HMGB1) and interferon-1 (IFN-I) were also investigated in patients with NSCLC whom have been treated with radiotherapy alone or in combination with immunotherapy (Ipilimumab), respectively. Whilst HMGB1 was not associated with survival rates, IFN-I showed to be predictive for treatment response (41).

### 2.3 Soluble receptors and ligands released from immune cell surface

Soluble receptors and ligands may also have potential predictive or prognostic value in patients with lung cancer treated with RT. Soluble receptors either are formed by alternative mRNA splicing, resulting in a polypeptide lacking a transmembrane region that is secreted by the cell, or it is a direct derivative from proteolytic cleavage of the membrane-bound receptor proteins from the cell surface (120). Receptors generally consist of a cytoplasmic domain, a transmembrane domain, and an extracellular domain. Soluble receptors generally comprise the extracellular domain and, therefore, retain the ability to bind the ligand (121). However, in contrast to membrane-bound receptors, soluble receptors cannot transmit signals to cells directly, but they can affect binding and activation of membrane receptors and co-receptors and, therefore, indirectly regulate cellular signaling (122). Recent studies have shown that circulating soluble receptors and ligands are potential cancer biomarkers, implicated in cancer progression, metastasis, immune evasion, and inflammation (44, 122, 123).

#### 2.3.1 Soluble tumor necrosis factor receptor-1

As mentioned before, TNF-α is an important pro-inflammatory cytokine involved in many pathologies (124). It exerts its biological effects by binding to receptors like TNFR1, which is widely expressed and, upon activation, can promote the proliferation, apoptosis or metastasis of lung cancer cells (125, 126). Soluble TNFR1 (sTNFR1) is generated by proteolytic cleavage of membrane-bound receptors by TNF-α converting enzyme (TACE), leading to a transiently reduced cellular responsiveness (127, 128). Interestingly, Hinton et al. showed that patients with stage II-IV NSCLC have higher baseline sTNFR1 before chemoradiotherapy compared to healthy individuals. Also, a temporary decline (2–4 weeks during RT) of sTNFR1 and TACE levels were observed in patients with RILI (59). Furthermore, Wang et al. showed that increased sTNFR1 levels 8 weeks post-chemoradiation were positively associated with increased symptom burden (e.g., pain, fatigue, distress) in 62 patients with stage I-IV NSCLC (42).

#### 2.3.2 Soluble interleukin-2 receptor

Interleukin-2 (IL-2) is a well-studied cytokine with pleiotropic effects (129). It can induce the activation of effector T cells and stimulate the growth of NK- and B-cells (130). IL-2R is expressed on various immune cells, varying from antigen-presenting cells to conventional T-cells and regulatory T-cells (Treg), releasing soluble IL-2 receptor (sIL-2R) upon immune activation (131). sIL-2R can modulate the biological function of IL-2 in serum (132). Carvalho et al. showed that higher concentrations of sIL-2R before start of treatment were associated with a worse OS in 181 patients with inoperable NSCLC who had undergone (chemo)-radiotherapy (43).

#### 2.3.3 Soluble programmed cell death ligand 1

Soluble forms of immune checkpoints have also been identified as key modulators in cancer pathogenesis (133). The PD-1/PD-L1 pathway controls the induction and maintenance of immune tolerance within the TME, with PD-L1 binding to PD-1 on T-cells to inhibit the immune response (134). PD-L1 is mainly expressed on tumor cells and some immune cells under inflammatory conditions (135). The soluble form of PD-L1 (sPD-L1) is produced by shedding the transmembrane domain of PD-L1 (136). Zhao et al. showed that high baseline levels of sPD-L1 were correlated with worse OS in patients with inoperable NSCLC treated with RT (44). They also showed that sPD-L1 levels tended to decrease during RT and got back to baseline levels months after RT. Similarly, Sui et al. reported in 31 patients with unresectable NSCLC that low levels of sPD-L1 before treatment initiation were associated with an objective response to concurrent chemoradiotherapy (45).

#### 2.3.4 Other soluble receptors and ligands

Other soluble receptors that were found to be related to treatment outcomes of patients with lung cancer treated with RT included CD244 and complement receptor 2 (CR2). CD244 (2B4) is a Signaling Lymphocyte Activation Molecule (SLAM) family immunomodulatory receptor that binds to high-affinity ligand CD48. CD244 is expressed by immune cells, such as monocytes, dendritic cells, NK cells, and T cells (137). Pre-clinical studies have shown that increased CD244 expression in the TME corresponds to increased immunosuppression via CD8+ T-cell exhaustion and increased production of immunosuppressors by MDSCs (138). Vaes et al. revealed that higher plasma CD244 levels before the first fraction of SBRT tended to be associated with a worse PFS in patients with stage I NSCLC (37). CR2 (Complement Receptor 2, or CD21) is a glycosylated transmembrane protein mainly expressed on B cells that binds to C3d, involved in linking the innate and adaptive immune system (139). Vaes et al. also showed that in patients with stage III NSCLC treated with concurrent chemoradiotherapy, higher levels of CR2 before the first fraction of RT were significantly associated with a better PFS (37).

### 2.4 Proteins on the immune cell surface of circulating immune cells

#### 2.4.1 Cluster of differentiation molecules

Naive- and effector CD8+ (cytotoxic) T cells are crucial in immune surveillance and the adaptive immunity against infection and cancer (140). The predictive and prognostic value of various CD8+ T-cell subsets have already been shown. Kim et al. indicated that high levels of T-cells with a senescence phenotype (CD57+CD28−CD8+ T cells) before, during and after treatment are correlated with increased RILI in 54 patients with stage II-III NSCLC treated with concurrent chemoradiotherapy. Also, high levels of circulating CD57+CD28−CD8+ T-cells before treatment were an independent predictor of grade ≥2 RILI (60). Besides, Liu et al. indicated that high amounts of CD8+CD28+T cells before treatment are related to improved early response to SABR in patients with metastatic NSCLC (46). In addition, Zafra and associates investigated CD8+PD1+ and CD8+PDL1+ as predictive biomarkers after the first stereotactic ablation RT fraction (141). However, both were elevated in the responders and non-responders, making them not eligible as distinct biomarker.

#### 2.4.2 T cell receptor repertoire

T-cell receptors (TCRs) are highly diverse heterodimeric surface receptors that mediate T-cell responses by recognizing specific antigens on major histocompatibility complex (MHC) molecules of antigen-presenting cells (APCs) (142). The spectrum of TCR epitopes responsible for tumor neoantigen recognition is diverse owing to the random formation of neoantigens, derived from numerous genetic alterations between patients (143). Each patient’s immune system must maintain a diversified TCR repertoire to recognize the variety of tumor neoantigens (144). Recent studies emphasize the role of TCR sequencing and repertoire analysis in understanding tumor biology, immune responses during treatment, and developing immunotherapies (145, 146). Increasing evidence indicated that the TCR repertoire changes after RT in patients with lung cancer (41, 147, 148). For example, Wu et al. indicated that the TCR repertoire diversity is reduced in patients with stage I NSCLC treated with SBRT (n=19). Moreover, diversity levels of TCR clones were lower after SBRT in the patients who developed distant metastases than in those who did not (47). Also, Öjlert et al. performed T-cell receptor sequencing in patients with stage IV NSCLC treated with SBRT and anti-PD-L1 (atezolizumab) immunotherapy (n=15) and showed decreased or stable diversity after RT in the best responders, and increased diversity at disease progression. Moreover, expansion of TCR clones was observed more often in responders (48). Similar results were reported by Formenti et al. in patients with metastatic lung cancer treated with CTLA-4 blockade combined with RT (41).

## 3 Discussion

**Figure 1:**
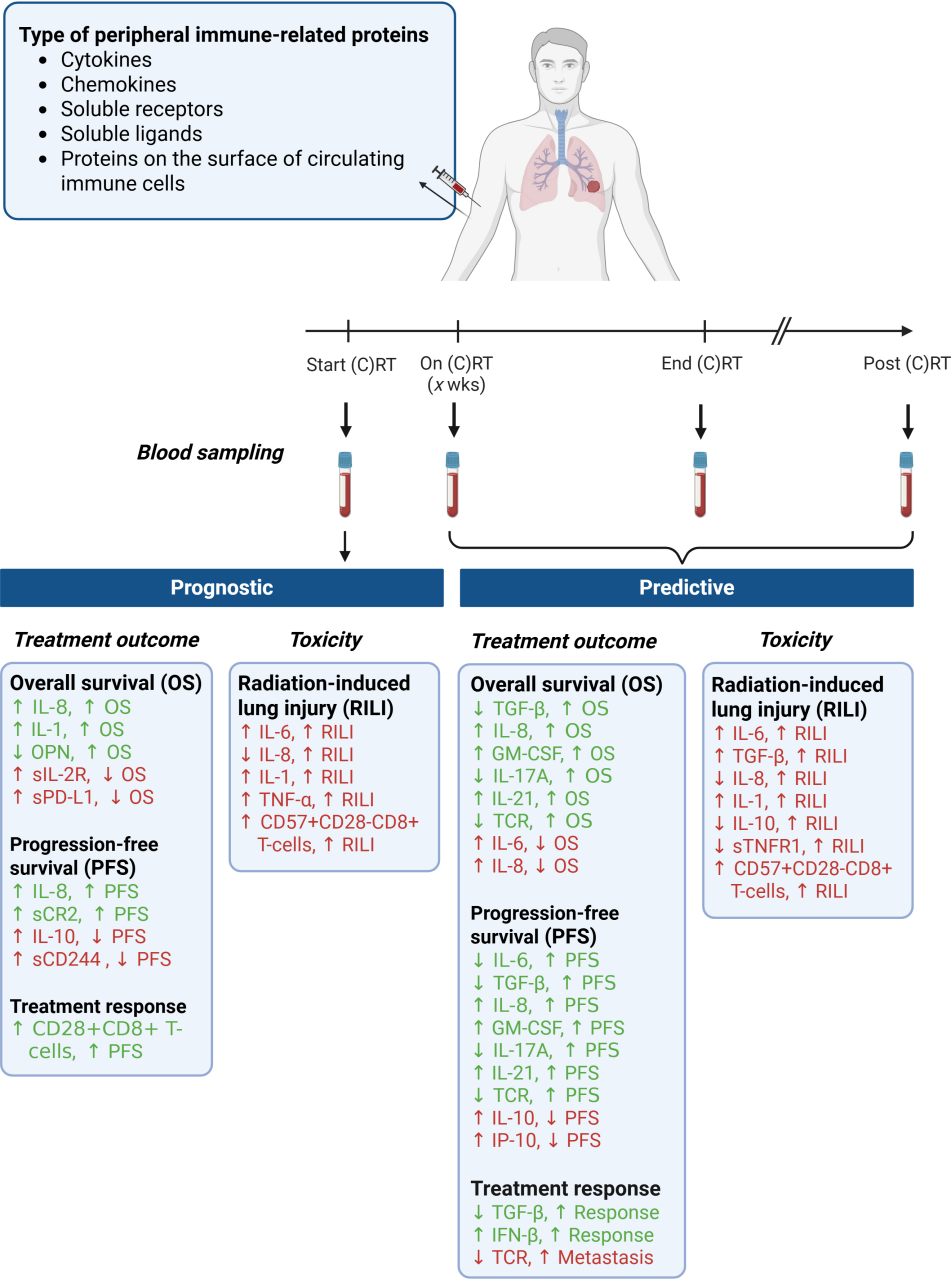
Prognostic and predictive biomarkers of peripheral immune-related proteins in patients with lung cancer treated with (chemo) radiotherapy. (C)RT, (chemo) radiotherapy; OS, overall survival; PFS, progression-free survival); RILI, radiation-induced lung injury. Created with BioRender.com.

The immune system’s diversity and the actual heterogeneous immune status before, during, and after treatment result in different treatment responses among patients. A reliable biomarker or preferably a panel of biomarkers to predict and monitor treatment responses is crucial for adjusting treatment protocols and personalizing interventions. This review summarized the current literature on peripheral immune-related molecules that may have prognostic and/or predictive value for outcomes and RT-induced toxicity in patients with lung cancer (**Figure 1**). Key molecules with a strong negative predictive value for treatment outcomes include IL-6, IL-8, and TGF-β1. Also, we observed that IL-6 and TGF-β1 are both prognostic and predictive for the development of RILI in patients with lung cancer. Other molecules, including IL-1, OPN, IP-10/CXCL10, IL-10, GM-CSF, IFN-β, IL-17A, IL-21, TNF-α, sTNFR1, sIL-2R, sPDL1, CD244, CR2, CD28+CD8+ T-cells, CD57+CD28-CD8 T-cells, and TCR repertoire have also been identified as potential prognostic or predictive biomarkers for patient outcomes. However, these molecules have only been assessed in a limited number of small-scale studies.

In this review, we showed that circulating levels of IL-6, IL-8, and TGF-β1 in the blood of patients with lung cancer treated with RT were consistently negatively associated with survival and RT-related toxicity. Measuring these proteins and validating their usefulness in real-life datasets will lead to improved patient selection and tailored preventive strategies for RT-induced toxicity. Besides, there are several ongoing phase 1/2 clinical trials aiming to investigate the clinical benefit of including blockade of IL-6, IL-8 or TGF-β in the treatment of patients with advanced lung cancer (IL-6: NCT05704634; IL-8: NCT04123379, NCT04572451; TGF-β: NCT03732274, NCT05537051). As such, IL-6, IL-8, and TGF-β are potential biomarkers and targets that should be further explored.

Despite extensive knowledge about the biological roles of these molecules, their clinical relevance as prognostic or predictive biomarkers remains poorly understood since their local biological effect is not always consistent with the clinically assessed responses. For example, IL-1 can induce inflammatory responses and can play a role in cancer progression, and as such, it was expected that IL-1 would negatively correlate with patient outcome (149). However, Tang et al. showed that higher baseline IL-1α levels were associated with improved outcomes in patients with oligometastatic NSCLC, possibly due to a systemic anti-tumor inflammatory state exhibited by induction chemotherapy (36). Similarly, it has been shown that irradiation can induce the expression of PD-L1 on the tumor cell surface of lung cancer cells (150). However, Zhao et al. showed that circulating sPD-L1 levels tended to decrease during RT and normalized 3 months after RT (44). This might be a result of the tumor death occurred by treatment and the recovered level of sPD-L1 coming from the immune cell response to RT. Also, IL-8, which is a pro-inflammatory cytokine, can stimulate collagen synthesis and matrix production inducing lung fibroblasts (97). However, in contrast to these local biological functions, low circulating levels of IL-8 before and during RT were associated with a higher risk of RILI in patients with NSCLC (52). These inconsistent findings highlight the need for more research and prospective clinical trials to understand better the mechanisms and clinical implications of these markers in RT.

Peripheral immune-related biomarkers are promising in the field of RT and can provide information on the host’s actual immune status before, during, and after treatment. Also, blood-based biomarkers can be monitored longitudinally and multiple biomarkers can be assessed simultaneously without sample limitation. Nevertheless, to date, there are still no reliable prognostic or predictive blood-based biomarker(s) to predict treatment outcomes in patients with lung cancer treated with RT, despite the circulating levels of numerous potential biomarkers have been assessed in several clinical trials.

An emerging but still incompletely understood phenomenon in the context of radiotherapy combined with immunotherapy is the abscopal effect - a systemic antitumor response occurring beyond the irradiated field (151). This effect has attracted growing interest as a potential outcome measure in immuno-oncology studies (29, 35, 41, 141, 152). However, current literature on the abscopal effect remains limited and presents several challenges. To date, only Mathew and his colleagues reported that abscopal responses (9 out of 29 patients) at 4 weeks following treatment were associated with prolonged increase in dendritic cells subset DC1, T-helper 1-like CD4 T-cells and circulating IL-12 (152). Other studies only report indirect correlations between immune-related peripheral-blood markers and abscopal responses, using surrogate outcomes like survival outcomes or objective response rates – complete or partial responses evaluated in all sites of the disease (29, 35, 41, 141). Notably, even when considering all cancer types, the occurrence of the abscopal effect remains rare. A systematic review by Abuodeh et al. highlighted that only 46 cases of abscopal effects were reported between 1969 and 2014, amongst which only 3 patients with primary lung cancer (153). Aside from the limited population, there is also lack of standardized, quantifiable criteria for defining and assessing abscopal responses, making it hard to corroborate the results.

Even though it falls outside the scope of our review, we want to acknowledge the relevance of other molecular biomarkers with potential immunological effects that can be measured in blood, encompassing circulating tumor cells (154), ctDNA (155, 156), m(i)RNA (141) and exosomes (35). For future studies developing risk assessment models for RT in combination with immunotherapy, a broader spectrum of biomarkers in blood should be explored than solely immune-related molecules.

**Figure 2:**
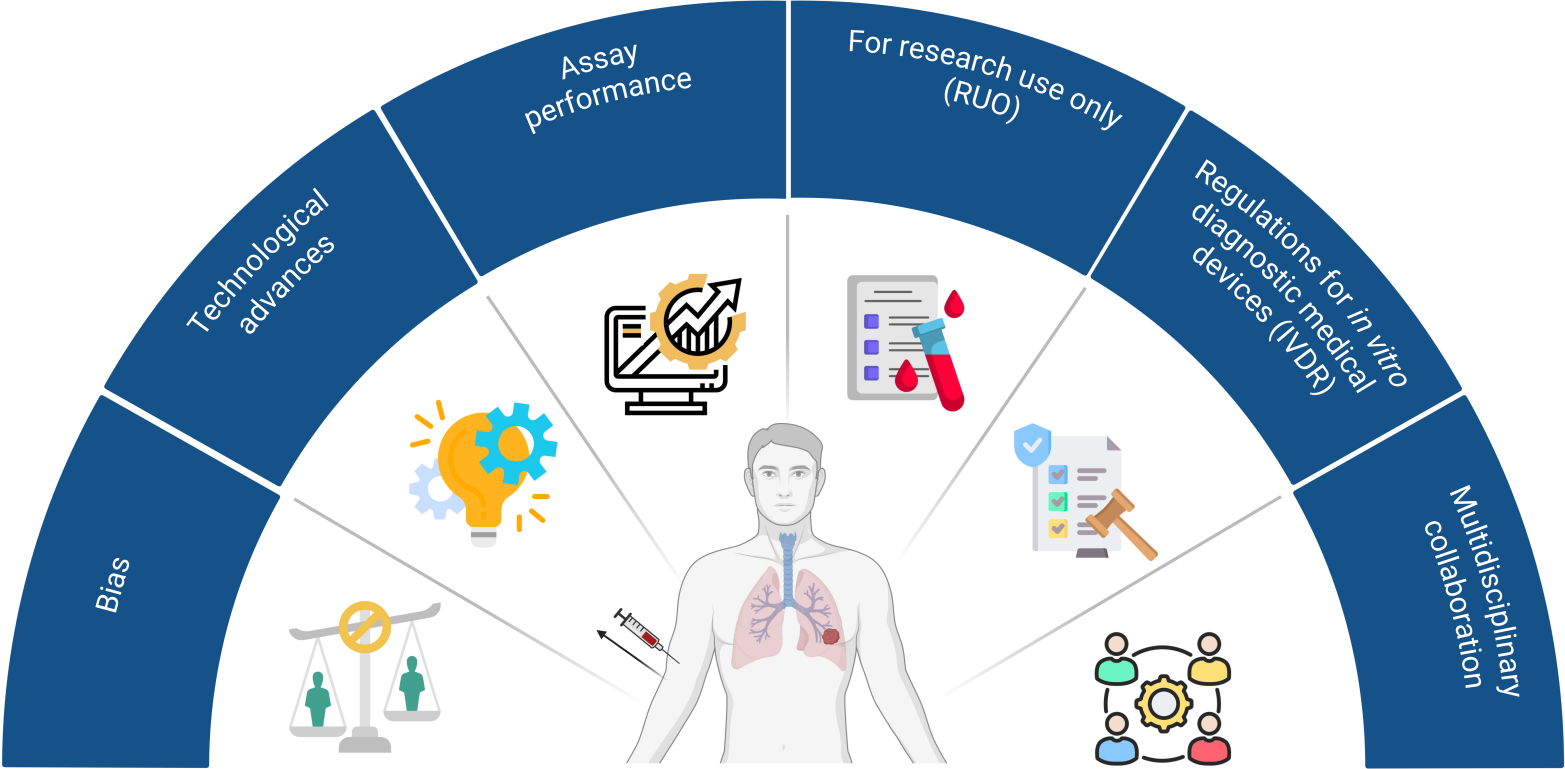
Current challenges and limitations to implement promising biomarkers in daily clinical care. 1) Bias is the main cause of failure in biomarker discovery and validation studies and can occur during patient selection, specimen collection, specimen analysis, and patient evaluation; 2) New technological advances have significantly enhanced the capacity for biomarker discovery, however, these innovations are expensive hampering the implementation thereof in large-scale studies; 3) Assays from different manufacturers vary in sensitivity and specificity. As a consequence, results from different trials cannot be compared. 4) Most assays are designated for research use only (RUO) and are not validated for diagnostic applications. 5) Manufacturers have to make strong investments (time, resources, and budget) to meet the new regulatory requirements to bring an *in vitro* diagnostic medical device (IVD) on the market (IVDR); 6) Lack of multidisciplinary collaboration among researchers, clinicians and industrial partners. Created with BioRender.com.

There are multiple challenges and limitations hampering both the identification and implementation of promising biomarkers in daily clinical practice (**Figure 2**). First of all, several types of biases causes failure in biomarker discovery and validation studies: for example, patient selection, specimen collection, specimen analysis, and patient evaluation. To date, numerous guidelines are available, providing researchers with an overview of practical considerations and potential pitfalls for their biomarker research (157, 158). Furthermore, recent technological advances have significantly enhanced the capacity for biomarker discovery, however, these innovations are often expensive. Consequently, many promising biomarkers remain confined to small-scale clinical studies, hampering the broader validation and clinical implementation. Also, the use of assays from different manufacturers further complicates the validation of promising biomarkers, while these vary in sensitivity and specificity. As a result, the outcomes from small-small scale studies cannot be compared. Also, most available tests are designated for research use only (RUO) and are therefore not validated for diagnostic applications. Lastly, the implementation of the ‘*In vitro Diagnostic Medical Devices Regulation’* (IVDR), which replaces the IVD Directive 98/79/EC, have resulted in drastic changes for practically all stakeholders (i.e. manufacturers, notified bodies, medical laboratories) in the field of *in vitro* diagnostic medical devices. As a consequence, manufacturers have to make strong investments (*i.e.* time, resources, and budget) to meet these new regulatory requirements (159, 160). Also, such regulations hinder the innovative capacity of medical laboratories preventing the development of new IVDs that could improve patient care.

Despite all these efforts, only a small number of biomarkers have been successfully validated and implemented in daily clinical care. One key barrier to clinical translation is the lack of multidisciplinary collaboration among researchers, clinicians, and industry partners. Most trials included in our review only assessed the circulating levels of specific proteins in a small sample size. However, due to significant heterogeneity in study designs, patient populations, and methodologies, the findings are difficult to compare and cannot be reliably corroborated. First, blood samples have been collected at different timepoints before, during and after treatment. Second, the patient populations vary substantially between the studies, including variations in tumor stages and treatment regimen (e.g., types of systemic drugs and various fractionation schemes). For example, low-doses radiation therapy have shown promising immunomodulatory effects, potentially reversing tumor resistance to immunotherapy, compared to high-dose radiation schemes (161). However, *in-vivo* evidence is currently lacking for lung cancer on the effect of different radiation schemes. As such, to set the stage for widespread clinical implementation and acceptance of these new lung cancer biomarkers, efforts are needed to set up collaborative, multidisciplinary studies.

## 4 Conclusion

This review focused on peripheral blood immune-related biomarkers with potential prognostic or predictive value for patients with lung cancer treated with RT. These findings could help researchers and clinicians to further validate promising candidates in prospective trials and implement them in daily clinical practice.
